# The Relationship between parental emotional support and geospatial thinking ability: a moderated mediating model

**DOI:** 10.3389/fpsyg.2026.1801735

**Published:** 2026-05-20

**Authors:** LingXuan Wang, Li Liu, Xue Meng, LeiLei Wang, Wei Ma

**Affiliations:** 1 School of Education, Shaanxi Normal University, Xi'an, Shaanxi, China; 2 College of Physical Education, Xi'an Physical Education University, Xi'an, Shaanxi, China; 3 College of Economics and Management, Huaibei Normal University, Huaibei, Anhui, China

**Keywords:** family structure, gender, geographic self-efficacy, geospatial thinking ability, parental emotional support

## Abstract

**Introduction:**

This study aims to clarify how parental emotional support affects high school students' spatial thinking ability.

**Methods:**

A quantitative survey was conducted to assess high school students' perceived parental emotional support, geographic self-efficacy, and geospatial thinking ability. Data were analyzed to examine the mediating role of geographic self-efficacy and the moderating effects of family structure and gender.

**Results:**

The results show that parental emotional support positively impacts geospatial thinking ability. Geographic self-efficacy mediates the relationship between parental emotional support and geospatial thinking ability. Single-parent families weaken the positive influence of parental emotional support on geospatial thinking ability. Female students have a positive moderating effect on the relationship between geographic self-efficacy and geospatial thinking ability.

**Discussion:**

This study expands existing research on the familial mechanisms influencing geospatial thinking ability and offers insights for geospatial thinking training. The findings underscore the importance of parents—particularly single parents—providing increased emotional support to students, while also emphasizing the need to encourage female students.

## 1 Introduction

The widespread application of geographic information technology in the Information Age has significantly increased the demand for geospatial thinking abilities, marking them as crucial for modern survival (Lee and Bednarz, 2009). Particularly relevant to high school students, geospatial thinking abilities have been linked to success across various STEM fields (Hegarty et al., 2010), serving as a cornerstone for numerous scientific disciplines and professions, thereby predicting higher academic and professional achievements (Kell et al., 2013; Kozhevnikov et al., 2007). Despite their crucial role in personal development, these abilities have yet to be emphasized within educational instruction.

In recent decades, the focus on geospatial thinking abilities has expanded across disciplines such as education, geography, psychology, and the learning sciences. Geospatial thinking ability involves the use of spatial thinking for spatial cognition, analysis, and reasoning within the context of the Earth's environment (Carbonell-Carrera et al., 2020). Many efforts have attempted to identify the factors influencing geospatial thinking ability to provide a theoretical basis for promoting their development. Influential factors on geospatial thinking ability are numerous, including personal factors like intelligence, neurological aspects, personal interests, gender, educational level, learning styles (Erskine et al., 2022; McGee, 1979; Xie et al., 2021), and external environmental factors such as societal, school, and family environments (Golledge and Stimson, 1997; Kim and Bednarz, 2013; Wei et al., 2022), all significantly impacting geospatial thinking ability.

Among these factors, family plays a pivotal role in shaping students' geospatial thinking abilities (Clingan-Siverly et al., 2021). Studies have found that the positive impact of family education and environment on spatial thinking levels (Harju-Luukkainen et al., 2020). Parents, in particular, may affect their children through various mechanisms, including both material aspects brought by their socioeconomic base and the immaterial aspects of emotional support. Existing research has indicated that parents' socioeconomic capital has a significant impact on children's geospatial thinking ability (Zhang et al., 2022). While increasing studies recognize the critical role of parental emotional support in children's psychosocial development (Boudreault-Bouchard et al., 2013; Pettit et al., 2011), few have focused on its impact on geospatial thinking ability.

Social cognitive theory posits a causal interrelation among environment, individual, and behavior, where self-efficacy is influenced by the individual's environment and, in turn, significantly affects individual behavioral motivation (Bandura, 1986). Recent research has elucidated the intricate connection between parental emotional support and various dimensions of a child's self-efficacy, encompassing career, entrepreneurial, and scientific self-efficacy (Lyons et al., 2023; Mao et al., 2017; Michael et al., 2013; Scott and Mallinckrodt, 2005). This body of work underscores the pivotal role of parents in the development of self-efficacy among adolescents (Turner and Lapan, 2002). Moreover, studies have shown that the spatial self-efficacy of students exerts a significant impact on their spatial thinking proficiency (Safadel et al., 2023). Relevant studies hint at a conceivable linkage between parental emotional support, geographic self-efficacy, and geospatial thinking abilities. Therefore, the predictive role of parental emotional support and its mechanisms on geospatial thinking ability warrants further investigation.

**Figure 1:**
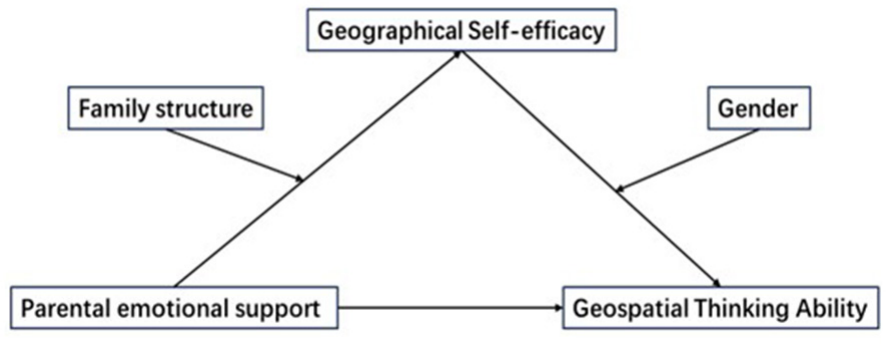
Theoretical model diagram.

The study will examine how parental emotional support affects geospatial thinking abilities, considering geographic self-efficacy as a mediator variable and family structure and gender as moderating variables, while controlling for residence (Figure 1). Comprehensive data collection was carried out across nine provinces in China to empirically ascertain the existence and impact of these mediating and moderating dynamics. The primary objective of this study is to delve into the intricate interplay between parental emotional support and children's geospatial thinking ability, with the aim of urging parents to invest greater emotional support in fostering their children's spatial cognition skills. Additionally, it emphasizes the significance of focusing on the spatial thinking development of students from single-parent family structures and female students during daily geospatial thinking training.

## 2 Literature review

### 2.1 Parental emotional support and geospatial thinking ability

According to the ecosystem theory, there is an interaction between individuals and their environment, with the family environment subsystem playing a crucial role in individual development (Gao et al., 2020). Family factors also significantly influence teenagers' geospatial thinking ability. The impact of family socioeconomic status on students' geospatial thinking abilities in geography has been studied (Zhang et al., 2023). However, compared to material factors, emotional characteristics of parental support such as understanding, care, respect, and recognition of children's expressions have a more profound effect on adolescents during periods of significant physical and cognitive changes (Shaw et al., 2004). Research has consistently demonstrated that emotional support from parents is strongly associated with higher levels of behavioral adaptation in adolescents (Zimmermann et al., 2008). Unconditional emotional support from parents represents the most beneficial form of support for adolescent development (Côté and Bouffard, 2011), as it effectively meets various needs for skill development among children (Guay et al., 1999). Undoubtedly, the ability to think geospatially holds immense importance.

Spatial thinking ability encompass the cognitive ability to mentally visualize and solve problems within a spatial framework (Nielsen et al., 2011). Geospatial thinking ability, a subset of spatial thinking ability in geography, focuses on using appropriate geospatial concepts, tools, and processes to solve relevant geographic problems in the geographic environment (Verma, 2014). Although there is currently no unified definition of geospatial thinking ability, its significance in daily life, scientific inquiry, and education commands attention across disciplines (Wakabayashi and Ishikawa, 2011). It has become undeniably significant that geospatial thinking ability is an essential quality for contemporary survival and plays a significant role in personal development, particularly in the realms of STEAM fields (Gagnier et al., 2017). Notably, geospatial thinking ability can be enhanced through specific training and intervention (Duarte and Teodoro, 2020), making the exploration of factors affecting geospatial thinking ability crucial for targeted training and intervention.

Based on the analysis of existing literature, it is apparent that parental emotional support, having numerous beneficial effects on cognitive development during adolescence, is likely to similarly enhance the development of geospatial thinking abilities. Consequently, this study puts forward the subsequent hypothesis: Parental emotional support can positively predict geospatial thinking ability (H1).

### 2.2 The mediating role of geographic self-efficacy

The attachment theory posits that the parent-child relationship's quality serves as the fundamental basis for parent-child attachment (Brumariu and Kerns, 2010). Emotional security derived from positive parent-child relationships facilitates children in developing a favorable perception of their own capabilities (Waters and Cummings, 2000). Children's perceptions of their abilities, influenced not only by age factors but also by their parents' influence (Harter, 1996), show that perceived parental support is positively correlated with children's self-perceptions of their abilities (Ohannessian et al., 1998). Especially, children who perceive a greater amount of emotional support from their parents tend to be more confident and positively approach problems when faced with difficulties, thereby enhancing their self-efficacy in dealing with challenges (Cohen and Wills, 1985). Emotional support can potentially enhance self-efficacy by fostering a sense of stability, bolstering self-esteem, and cultivating a perception of control (Deborah et al., 2017).

In the specific domain of geography, self-efficacy pertains to the extent of an individual's belief in their competence to apply geospatial knowledge and successfully accomplish relevant spatial tasks (Mitolo et al., 2015). In the field of spatial cognition, the findings of previous research have demonstrated a strong correlation between spatial self-efficacy and spatial abilities (Safadel et al., 2023). Some argue that spatial self-efficacy can predict performance in finding shortcuts (Pazzaglia et al., 2016). Given geography's intrinsic connection to spatial elements, it is inferred that geographic self-efficacy might share a significant relationship with geospatial thinking abilities, although this correlation has not been independently explored within the domain of spatial cognition.

In light of these precedents, we introduce the following hypothesis: Parental emotional support indirectly influences high school students' geographic self-efficacy, which in turn impacts their geospatial thinking abilities (H2).

### 2.3 The moderating role of family structure

Adolescence represents a crucial phase marked by significant physical, psychological, and social transitions, necessitating specific mental and physical health needs (Casey et al., 2010). The family environment represents the closest social context for adolescents, and variations in family structure play a crucial role in understanding the heterogeneity of adolescent experiences (Wikle and Hoagland, 2020). According to relational regulation theory, social interactions exert regulatory effects on emotions, thoughts, and behaviors among adolescents (Rogers et al., 2018). Interactions with individual families can influence emotional responses in adolescents while different family structures may have varying emotional consequences (Kim et al., 2018; Elliott et al., 2016; Wikle et al., 2019).

Research has demonstrated that children within intact families tend to receive greater emotional support and encouragement (Lam et al., 2012), fostering higher levels of self-efficacy. Notably, there exists a strong association between family structure and adolescent behavior as well as health issues (Magnuson and Berger, 2009). Establishing positive parent-child relationships may prove more challenging within reconstituted families compared to biological families (Hetherington, 1999), potentially leading to increased stress among adolescents which further diminishes their ability to develop optimal self-efficacy levels (Turunen, 2013). Single-parent family structures often face greater parenting stress (Berryhill, 2016), with specific vulnerabilities like low socioeconomic status and strained parental relationships increasing the likelihood of adverse risks and lower wellbeing for children (Wu et al., 2008).

The detrimental effects on adolescent development within single-parent and reconstituted families are often attributed to diminished parental supervision and engagement (Arnold et al., 2017). Studies indicate that the dissolution of partner relationships and the formation of reconstituted families may weaken the bonds between children and their parents (Moor and Komter, 2012). However, parental support, especially in its emotional form, can alleviate some of the psychological and wellbeing risks associated with specific family configurations (Patten et al., 1997). Evidence suggests that positive parent-child relationships correlate with enhanced academic performance and motivation among adolescents (Morrison et al., 2003). Furthermore, these relationships can act as a buffer against risky behaviors and mental health challenges in youth (Qu et al., 2015). However, single-parent and reconstituted families often grapple with varying levels of parent-child relational crises (Coleman and Ganong, 1990), potentially impacting the extent of emotional support perceived by the child.

Thus, drawing from the above literature review, we propose the hypothesis: The moderating role of family structure on the mediating effect of parental emotional support in relation to geographic self-efficacy (H3).

### 2.4 The moderating role of gender

In recent explorations of spatial thinking abilities, individual differences, notably those associated with gender, have increasingly captured the attention of scholars (Ishikawa, 2021). In regards to disparities in spatial thinking ability between genders, research findings consistently indicate significant disparities between males and females in terms of performance (Abad et al., 2018). This is particularly evident in components of spatial ability like mental rotation tests, where females consistently score lower than males (Hegarty, 2018). However, studies on minors do not consistently show gender differences in spatial thinking abilities. A study on spatial visualization from Kuwait revealed boys' superior performance over girls in spatial visualization tests (Nazareth et al., 2013). Investigation into gender differences in spatial reasoning among young children indicated that boys outperformed girls only after training; absent additional training, no significant gender differences were observed in spatial reasoning (Joh, 2016). Similarly, assessments of young children's spatial thinking abilities across various tasks found no notable gender differences (Verdine et al., 2017). Interestingly, even in the domain of mental rotation, where adult findings are more consistent, research on young children has identified no significant gender differences (Frick et al., 2013). Obviously, further research is needed on gender differences in spatial thinking abilities.

Prior studies have demonstrated that spatial thinking skills exhibit diverse results among different age cohorts when considering gender disparities. Some scholars have proposed that this phenomenon is associated with the socialization process influenced by parents and the early childhood self-assessment of spatial thinking abilities. Regarding the socialization process, due to the impact of stereotypical gender attitudes, parents tend to interact differently with boys and girls, potentially fostering boys' spatial ability and girls' social ability (Wang and Sui, 2019). Concerning self-assessment of geospatial thinking ability, self-efficacy exerts a significant influence on such evaluations. Studies on spatial self-efficacy confirm that girls have lower spatial self-efficacy than boys, despite sometimes demonstrating superior actual performance (West et al., 2002). Consequently, men tend to overestimate their spatial thinking abilities while women tend to underestimate theirs when reporting their performance in spatial navigation tasks (Condon et al., 2015). Through the aforementioned literature review, we propose that the moderating role of gender may influence the mediating effect of geographic self-efficacy on geospatial thinking ability (H4).

## 3 Method

### 3.1 Participants

This research targeted first-year high school students in China, drawing participants from nine public high schools across the eastern, central, and western regions of the country, the combined student population amounts to 1,102 individuals. The survey was conducted through paper questionnaires distributed and completed under teacher supervision during geography classes. It comprised two parts: the first questionnaire included demographic characteristics (gender; family structure; residence), a parental emotional support survey, and a geographic self-efficacy survey, while the second consisted of a geospatial thinking ability test. Valid responses were obtained from 862 participants for the first questionnaire and 1,060 for the second, with those completing both questionnaires retained for the study sample (*N* = 862, retention rate of 78.22%). The final sample comprised 52.90% females (*n* = 456) and 47.10% males (*n* = 406). The Ethics Review Committee of the University, to which the researcher is affiliated, granted ethical approval for the questionnaire. Furthermore, written informed consent was obtained from both participants and their parents. In order to ensure privacy protection, student-completed questionnaires were anonymized.

### 3.2 Measures

The study employed four data components: demographic information, questionnaires on parental emotional support, a scale measuring geographic Self-Efficacy, and a test assessing geospatial thinking ability. The demographic information included the participant's gender, family structure, and place of residence. Gender was categorized as male or female; family structure referred to the composition of family members and their interrelationships, which were categorized as intact families (where the child lives with two biological or adoptive parents), reconstituted families (where the child lives with father and stepmother or mother and stepfather), and single-parent families (where the child lives with a father/mother or other relatives). Residence is classified into urban areas and rural.

#### 3.2.1 Parental emotional support

This questionnaire was adapted from the 2018 Program for International Student Assessment's (PISA) No. 123 questionnaire on student' perceived parental emotional support. Emotional support is defined as the extent to which interpersonal relationships are perceived as intimate, trustworthy, and fulfilling (Slavin and Rainer, 1990). Parental emotional support refers to the emotional attributes exhibited by parents during the process of nurturing children, such as parental attentiveness, emotional expression, and positive reinforcement (Huver et al., 2010). Designed to assess these emotional characteristics from the child's perspective, the scale comprises 3 questions with positive statements, including: “My academic pursuits and accomplishments receive unwavering support from my parents,” “When encountering challenges in my academic pursuits, I receive unwavering support from my parents.” and “The encouragement from my parents fosters the development of my self-confidence.” The responses were assessed using a 4-point Likert scale (1 = strongly disagree, 4 = strongly agree), with total scores ranging from 0 to 12. Higher scores denote greater perceived parental emotional support. In this research, the Cronbach's α coefficient for this scale was found to be 0.90, and the McDonald's ω coefficient was also determined to be 0.90, indicating high reliability.

#### 3.2.2 Geographic Self-Efficacy

Adapted from the PISA 2012 No. 30 questionnaire on mathematics self-efficacy, this scale measures beliefs, judgments, or subjective feelings regarding one's capability to execute tasks using personal skills, as conceptualized by Bandura (2012). Bandura (2012) also noted the importance of analyzing the core structures and content of a specific domain to construct an appropriate domain-specific self-efficacy scale. The essence of geography lies in the comprehensive examination of spatial analysis, interactions between humans and their environment, location-based analysis, and regional analysis (Baerwald, 2010). The geographic self-efficacy questionnaire is designed to assess students' confidence in mastering these core contents, with 8 questions such as, “I have the ability to select the most suitable site by considering provided spatial characteristics, including land utilization, altitude, and population concentration, and superimposing data related to these factors,” and “I can envision the visual representation of certain shapes (triangles, squares, etc.) undergoing rotation, distortion, and overlapping” The 4-point Likert rating system was utilized in this study, ranging from 1 (indicating no confidence) to 4 (indicating high confidence), to measure the level of geographic self-efficacy. The total scores on this scale ranged from 0 to 32, with higher scores indicating a stronger sense of geographic self-efficacy. Reliability analysis demonstrated that the scale exhibited high internal consistency, as evidenced by a Cronbach α coefficient of 0.90 and a McDonald ω coefficient of 0.89.

#### 3.2.3 Geospatial thinking ability

The Spatial Thinking Ability questionnaire presented here is derived from the work of Lee and Bednarz (2012). According to the National Research Council (NRC, 2006), the spatial thinking ability encompasses three main components: the characteristics of space, the methods used to represent spatial information, and the cognitive process involved in spatial reasoning. Lee and Bednarz (2012) utilized this definition to develop their test, covering abilities such as understanding direction, identifying maps, spatial selection, visualizing slope profiles, recognizing spatial correlation (positive or negative), dimension conversion, selecting images, and geographic analysis. The original purpose of its creation was to evaluate the impact of GIS education on geospatial thinking abilities, but it has since been employed for a broader assessment of students' spatial thinking abilities (Collins, 2018). Recognized as a reliable tool for assessing spatial thinking abilities (Bednarz and Lee, 2019), the test consists of 16 multiple-choice questions, yielding scores ranging from 0 to 16. The higher the scores, the stronger the geospatial thinking abilities. The Cronbach α coefficient for this scale in the study was found to be 0.79, while the McDonald ω coefficient yielded a value of 0.84, indicating high levels of reliability.

### 3.3 Data analysis

Firstly, we conducted a common method variance analysis on the research data. The results of the Hair single-factor test revealed three factors with eigenvalues greater than 1. However, the first factor accounted for only 33.80%, below the 40% threshold, indicating no common method bias and permitting further data analysis. Secondly, we employed Pearson correlation coefficients to assess the linear association between parental emotional support, geospatial thinking ability, and geographic self-efficacy. Finally, Mplus8.3 software was utilized to examine mediating and moderating paths between variables in order to confirm all proposed hypotheses in this research.

## 4 Results

### 4.1 Confirmatory factor analysis and validity

We conducted confirmatory factor analysis (CFA) to evaluate the validity of the study's factor structure. Considering that family structure and gender are single-question measured variables as moderating factors, only Parental Emotional Support Scale, Geographic Self-Efficacy Scale, and the Geospatial Thinking Abilities Scale were included in the CFA. As a result of weak loading (0.11), we decided to exclude the sixth dimension from the Geospatial Thinking Ability Questionnaire in our model. Following this adjustment, our model demonstrated excellent fit (CMIN/DF = 2.32, p < 0.001, CFI = 0.98, TLI = 0.97, RMSEA = 0.04, SRMR = 0.04). Moreover, we found that the Parental Emotional Support Scale exhibited high composite reliability (CR) value of 0.90 and average variance extracted (AVE) value of 0.74; the Geographic Self-Efficacy Scale displayed a CR value of 0.89 and an AVE value of 0.50; finally, the Geospatial Thinking Ability Scale showed a CR value of 0.83 and an AVE value of 0.48 (refer to Table 1). Additionally, the square root values for each factor's AVE exceeded their respective inter-factor correlations (refer to Table 2), indicating satisfactory discriminant validity for all Scales employed in this research. The overall assessment confirms that our study Questionnaires are considered acceptable.

**Table 1:** The test of validity.

Variables	Item	Item	Loading	AVE	CR
Factors1	Parental Emotional Support	PES 1	0.85	0.74	0.90
		PES 2	0.89		
		PES 3	0.84		
Factors2	Geographic self-efficacy	GSE 1	0.74	0.50	0.89
		GSE 2	0.72		
		GSE 3	0.73		
		GSE 4	0.68		
		GSE 5	0.69		
		GSE 6	0.75		
		GSE 7	0.62		
		GSE 8	0.74		
Factors3	Geospatial thinking ability	GTA 1	0.70	0.48	0.83
		GTA 2	0.66		
		GTA 3	0.48		
		GTA 4	0.59		
		GTA 5	0.54		
		GTA 7	0.76		
		GTA 8	0.73		

**Table 2:** Distinctiveness of variable validity.

Variables	Factor 1	Factor 2	Factors 3
Factor 1	0.87		
Factor 2	0.39	0.71	
Factors 3	0.40	0.40	0.69

### 4.2 Descriptive statistics and correlation analysis

According to the results in Table 3, correlation analysis revealed strong positive correlations among parental emotional support, geographic self-efficacy, and geospatial thinking abilities, which are variables used in the mediation analysis. These relationships support the follow-up hypothesis analysis.

**Table 3:** Statistical description and relational examination in the study.

Variables	Mean	SD	a	b	c
a. Parental emotional support	10.82	1.37	1		
b. Geographic self-efficacy	21.80	4.31	0.09^**^	1	
c. Geospatial thinking ability	12.27	3.35	0.09^*^	0.15^***^	1

N, 862. ^*^p < 0.05, ^**^p < 0.01, ^***^p < 0.001.

### 4.3 Mediation analysis

**Figure 2:**
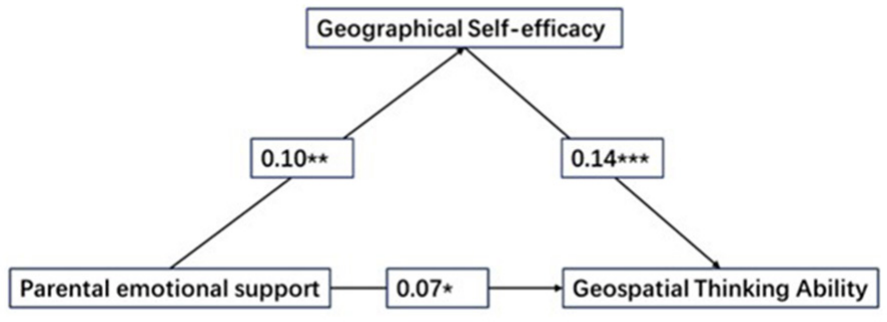
Mediation model with Geographic self-efficacy as the mediator.

Given the substantial correlations among parental emotional support, geographic self-efficacy, and geospatial thinking abilities, this study employs the Mplus8.3 to investigate the mediating role of geographic self-efficacy in the influence of parental emotional support on geospatial thinking abilities. Prior to analysis, a standard normalization process is applied to all data sets. The initial step involved assessing the overall impact of parental emotional support on geospatial thinking abilities, which reveals that such support significantly and positively influences geospatial thinking abilities (β = 0.09, *SE* = 0.03, *t* = 2.54, *p* = 0.011). By incorporating geographic self-efficacy as a mediator variable into the model, both the mediation framework (see Figure 2) and the outcomes of the mediation analysis (see Table 4) are obtained. The findings demonstrate that parental emotional support significantly and positively affects both geographic self-efficacy and geospatial thinking abilities (β = 0.09, SE = 0.03, t = 2.98, *p* = 0.003; β = 0.07, SE = 0.03, t = 2.12, *p* = 0.034), with geographic self-efficacy also showing a significant positive impact on geospatial thinking abilities (β = 0.14, SE = 0.04, *t* = 4.12, *p* < 0.001). Additionally, the bootstrap method is utilized to assess the confidence intervals (95%) for both the direct and indirect effects of parental emotional support on geospatial thinking abilities, with neither the upper nor the lower bounds containing zero, indicating that parental emotional support directly and indirectly predicts geospatial thinking abilities through geographic self-efficacy. The direct and mediated effects accounts for 84.22% and 15.78% of the total effect, respectively (see Table 5).

**Table 4:** The mediation influence of parental emotional support on geospatial thinking ability.

Variables	M: Geographic self-efficacy			Y: Geospatial Thinking Ability		
	β	*SE*	*t*	β	*SE*	*t*
Parental Emotional Support	0.09	0.03	2.98^**^	0.07	0.03	2.12^*^
Geographic self-efficacy				0.14	0.04	4.12^***^
Residence	−0.06	0.08	−0.77	−0.06	0.08	−0.72

N, 862. ^*^p < 0.05, ^**^p < 0.01, ^***^p < 0.001.

**Table 5:** The impact magnitude of mediation analysis.

Effect type	Impact magnitude	Percentage of impact degree
Total effect	0.09	100.00%
Direct effect	0.01	84.22%
Indirect effect	0.07	15.78%

### 4.4 Moderated mediation analysis

In order to determine whether family structure and gender act as moderating variables within the established mediation framework, this research investigated the influence of family structure on the connection between parental emotional support and geographic self-efficacy, in conjunction with the influence of gender on the connection between geographic self-efficacy and geospatial thinking abilities. As family structure and gender are categorical variables, they were virtually coded during data processing. In the moderation analysis for gender, males were used as the reference category, while intact families served as the reference for family structure analysis. As demonstrated in Table 6, the findings signify that family structure significantly moderates the impact of parental emotional support on geographic self-efficacy (β = −0.10, SE = 0.04, t = −2.60, *p* = 0.009; β = −0.32, SE = 0.06, t = −5.77, *p* < 0.001), and gender significantly moderates the impact of geographic self-efficacy on geospatial thinking abilities (β = 0.18, SE = 0.07, *t* = 2.66, *p* = 0.008). The impact of these moderating variables varies depending on their specific values (refer to Table 7). In samples with intact families and single-parent families, women significantly moderated the connection between parental emotional support and geographic self-efficacy. However, men did not exhibit such a significant role. In reconstituted family samples, no significant moderating effect was observed between parental emotional support and geographic self-efficacy for both men and women.

**Table 6:** The moderated mediating effect analysis regarding Parental Emotional Support and Geospatial Thinking Ability.

Variables	M: Geographic self-efficacy			Y: Geospatial Thinking Ability		
	β	*SE*	*t*	β	*SE*	*t*
Parental Emotional Support	0.07	0.03	7.96^*^	0.07	0.04	1.97^*^
Reconstituted family	−1.59	0.04	−39.78^***^			
Single-parent family	−2.32	0.08	−30.39^***^			
Parental Emotional Support x Reconstituted family	−0.10	0.04	−2.60^**^			
Parental Emotional Support x Single-parent family	−0.32	0.06	−5.77^***^			
Geographic self-efficacy				0.06	0.05	1.05
Female				−0.03	0.07	−0.45
Geographic self-efficacy x Female				0.18	0.07	2.66^**^
Residence	−0.08	0.06	−1.38	−0.07	0.08	−0.87

^*^p < 0.05, ^**^p < 0.01, ^***^p < 0.001.

**Table 7:** Indirect effect of mediating models under different conditions.

Category	Impact magnitude	Boot LLCI	Boot ULCI
Intact family			
Male	0.00	−0.00	0.02
Female	0.02	0.00	0.03
Reconstituted family			
Male	−0.00	−0.01	0.00
Female	−0.01	−0.02	0.00
Single-parent family			
Male	−0.01	−0.05	0.01
Female	−0.06	−0.10	−0.04

### 4.5 Simple slope analysis

**Figure 3:**
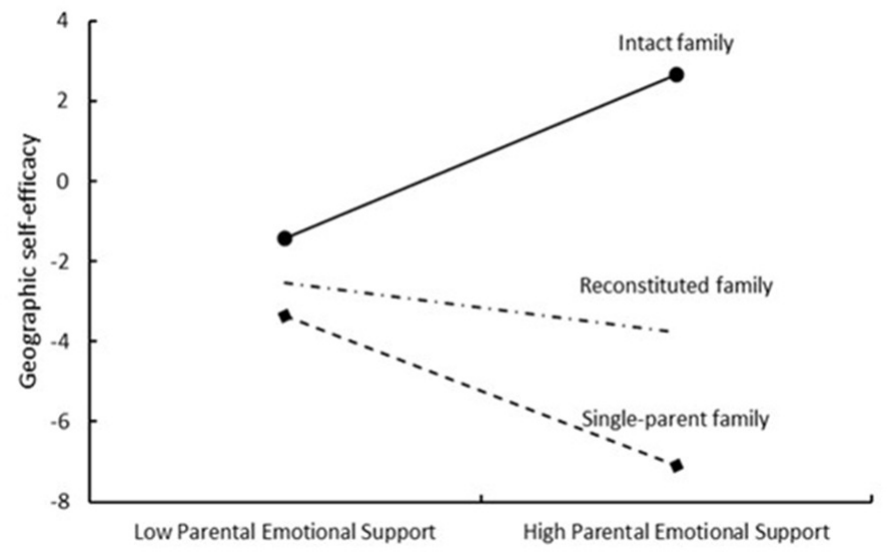
Moderating effect of family structure on the prediction of geographic self-efficacy from parental emotional support.

**Figure 4:**
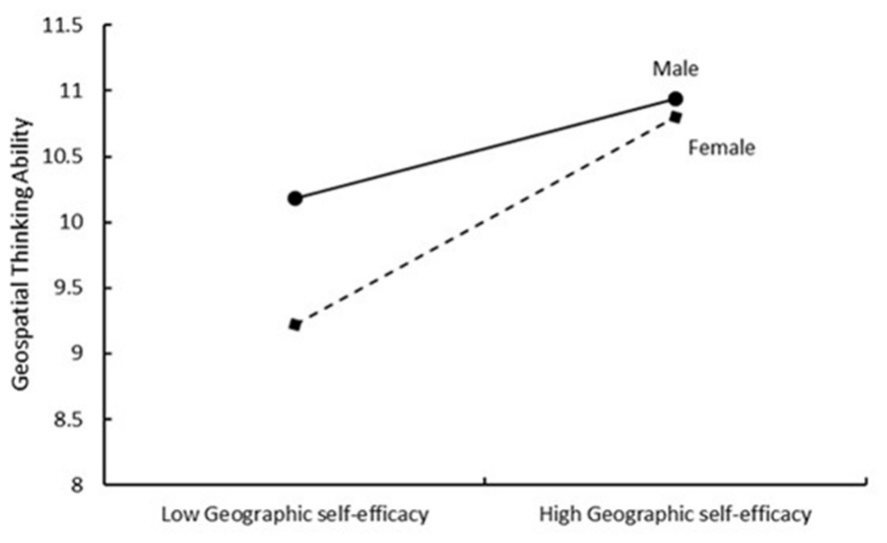
Moderating effect of gender on the prediction of geospatial thinking abilities from geographic self-efficacy.

Simple slope analyses (refer to Figure 3) reveal that within the intact family group, parental emotional support positively predicts geographic self-efficacy (β = 0.06, SE = 0.03, t = 2.18, p = 0.030). Conversely, in single-parent family groups, parental emotional support negatively predicts geographic self-efficacy (β = −0.26, SE = 0.05, *t* = −5.45, *p* < 0.001). The moderating influence of a reconstituted family on the linkage between parental emotional support and geographical self-efficacy did not yield statistically significant results (β = −0.04, SE = 0.03, *t* = −1.42, *p* = 0.157). According to Figure 4, for females, geographic self-efficacy positively predicts geospatial thinking abilities (β = 0.08, SE = 0.03, *t* = 2.69, *p* = 0.01), whereas for males, the moderating effect of geographic self-efficacy on the influence path of geospatial thinking ability was not found to be significant (β = 0.06, SE = 0.05, *t* = 1.05, *p* = 0.293).

Overall, the evidence lends strong support to the hypothesized model of moderated mediation outlined in this study.

## 5 Discussion

This research utilizes a moderated mediation framework to investigate the pathways by which parental emotional encouragement affects individuals' geospatial reasoning skills. Findings reveal that such support from parents fosters geospatial reasoning through the intermediary role of geographic self-confidence. Additionally, the study discovers that the beneficial impact of parental emotional encouragement on geospatial reasoning is diminished for students from single-parent households, and a similar reduction is observed in the positive role of geographic self-confidence on geospatial reasoning among female students. These findings corroborate the hypotheses posited earlier, thereby expanding the research on family influence mechanisms pertaining to geospatial thinking abilities. Additionally, this study confirms the heterogeneous roles of family structure and gender in this mechanism, offering insights into the emphasis on parental, particularly in single-parent families, support and the importance of fostering an encouraging educational environment for female students in geospatial thinking training.

### 5.1 The influence of parental emotional support on geospatial thinking abilities

The study indicates that parental emotional support positively predicts geospatial thinking abilities, aligning with prior studies that parental emotional support can fulfill children's needs for capability development to some extent (Deci and Ryan, 1975). According to the social cognitive model, parents play a crucial role in children's perception of their capability development (Bandura, 1989). When parents exhibit emotional support, such as encouragement and satisfaction, children's cognitive development of capabilities is imbued with positive significance (Galand and Vanlede, 2004). A study has shown a positive correlation between students' self-perceived abilities and their parents' satisfaction with their academic performance (McGrath and Repetti, 2000). Moreover, emotional support from parents is noted to increase as children transition into adulthood (Lefkowitz, 2005), suggesting that during the pivotal developmental stage of high school, parental emotional support critically influences children's self-assessment of their capabilities. Sociological studies highlight that parental emotional support, as an important family factor, is closely linked to adolescents' career development and competency-related skills (Whiston and Keller, 2004). It is noteworthy that while parental emotional support is acknowledged as a vital catalyst for adolescent capability development, it has been relatively underexplored, particularly concerning its impact on the development of geospatial thinking abilities. Our investigation into the effect of parental emotional support on the development of geospatial thinking abilities in high school students is exploratory and calls for further research to substantiate these findings.

### 5.2 The mediating role of geographic self-efficacy

We discover that geographic self-efficacy serves as a mediator between parental emotional support and geospatial thinking abilities, indicating that parental emotional support is not only directly related to high school students' geospatial thinking ability but also indirectly through students' geographic self-efficacy. According to attachment theory, parents play a crucial role in developing healthy attachment relationships, as the quality of parent-child interactions forms the foundation for developing internal working models (Bowlby, 1982). These models link various parenting styles experienced by children to the development of beliefs and expectations. High-quality parent-child relationships foster self-efficacy, leading to positive internal working models that significantly benefit children's development of abilities. the development of individual spatial thinking abilities is intricately linked to self-efficacy. In a study conducted by Cao et al. (2023), the incorporation of Web GIS into geography classroom instruction not only fostered students' ongoing engagement with geography but also effectively enhanced their self-efficacy and spatial thinking prowess. Moreover, Safadel et al. (2023) provided direct evidence indicating a positive link between spatial self-efficacy and spatial ability. Beyond its crucial role in spatial thinking ability development, self-efficacy's importance in critical and creative thinking has also been confirmed (Hyytinen et al., 2018; Redifer et al., 2021). Overall, the significant role of self-efficacy in thinking ability development has become a consensus among researchers. Therefore, incorporating more interventions related to geographic self-efficacy and geospatial thinking in geography education could significantly enhance high school students' geospatial thinking abilities.

### 5.3 The moderating role of family structure

Our study found that family structure moderates the direct effect of parental emotional support on geographic self-efficacy. Specifically, students from intact families experience an enhanced positive impact of parental emotional support on their geospatial thinking abilities, while those from single-parent families experience a diminished effect. This observation aligns with the majority of existing studies (Barrett and Turner, 2005; Daatland, 2007; Tomassini et al., 2004). One possible explanation could be material-based: single-parent families often face higher socioeconomic pressures compared to intact families, which can impede parent-child interactions (Crosnoe and Cavanagh, 2010) and set the stage for strained parent-child relationships, thereby increasing adolescents' risk for negative behavioral outcomes and unhealthy psychological states. Another explanation is from the psychological needs perspective, complex family relations and fragile communication environments in single-parent or reconstituted families hinder emotional connections between children and parents, resulting in emotional estrangement and attachment disruption (Shapiro, 2003). However, these findings are not universally consistent across different research contexts. Some studies have indicated that emotional bonds between mothers and children in divorced families may become stronger Cooney et al. (1986), suggesting that post-divorce parent-child relationships may improve due to compensatory psychological mechanisms. That is, the literature presents mixed findings regarding the impact of family structure on parent-child relationships. Some researchers suggest that operational models of different family structures may vary significantly due to diverse racial cultures, possibly explaining the differing conclusions in the literature (Cross, 2020).

### 5.4 The moderating role of gender

Our study indicates that gender acts as a moderator on the direct effect of geographic self-efficacy on geospatial thinking. Females exhibit a positive influence of geographic self-efficacy on geospatial thinking abilities, corroborating prior studies. Grounded in the theory of incremental intelligence, the belief in the malleability of intelligence and abilities significantly influences individual behavior and performance. Holding the view that intelligence and skills can be developed bolsters effort in learning and resilience in the face of challenges (Dweck and Yeager, 2019), a process underpinned by self-efficacy. Spatial thinking ability, a crucial aspect of intelligence (Thurstone, 1948), is significantly governed by such beliefs. Extensive research into spatial thinking abilities further underscores gender disparities (Boone et al., 2018; Munion et al., 2019; Voyer et al., 2017). It is plausible that gender moderates the influence of self-efficacy on spatial thinking abilities. Latest research suggests that gender differences in geospatial thinking ability may be more evident in self-evaluation and efficacy beliefs than in fundamental ability levels. Hofer et al. (2025) found that women reported significantly lower interest in STEM (Science, Technology, Engineering, and Mathematics) careers—and this interest was more strongly correlated with self-evaluated spatial intelligence than with objectively measured spatial intelligence. This implies that even when women and men have comparable objective abilities, women's underestimation of their own capabilities may undermine their willingness to pursue STEM fields. The influence of such stereotypes on self-perception of spatial ability can even be traced back to the preschool stage. Ebert et al. (2024) found that children aged 5-6 already hold implicit and explicit stereotypical beliefs that “spatial ability belongs to boys.” Together, this evidence points to a possible conclusion: behind the influence of geographical self-efficacy on geospatial thinking ability, it is more likely to be gender confidence differences shaped by stereotypes rather than innate gender differences in ability. It is noteworthy, however, that despite males demonstrating significant advantages in both spatial self-efficacy and spatial thinking abilities, some studies suggest that these advantages might be attributed to the assessment of specific task types that favor males (Miola et al., 2023). Conversely, when tasks align more closely with female strengths, females outperform males in spatial thinking abilities (Nori et al., 2018). In summary, while gender differences in spatial self-efficacy and spatial thinking abilities are widely recognized among scholars, arriving at a universally accepted conclusion regarding gender differences in spatial thinking abilities remains elusive, underscoring the need for further research.

## 6 Conclusion

To summarize our findings, this research confirms the proposed hypotheses and demonstrates that parental emotional support significantly predicts geospatial thinking ability. Geographic self-efficacy serves as a crucial mediator in this relationship. Moreover, family structure and gender are identified as important factors that influence the mediating effect of geographic self-efficacy. Specifically, it is observed that the favorable influence of parental emotional support on spatial thinking abilities is weakened for students from single-parent families, while female students positively moderate the link between geographic self-efficacy and spatial thinking ability. Therefore, emphasizing the importance of parental emotional support in geospatial thinking abilities education is essential, especially for those from single-parent families. Additionally, tailoring educational strategies to better support female students is imperative.

## 7 Limitation and implication

Despite its merits, this research has its limitations. First, the cross-sectional design of this study limits its ability to capture the nuanced relationship between parental emotional support and geospatial thinking abilities in specific contextual settings. Future research could consider adopting a longitudinal approach to explore how parental emotional support influences geospatial thinking abilities over an extended period of time. Second, the potential impact of our findings may be limited in terms of generalizability due to the specific focus on adolescents at the high school level. The scope of future research could be broadened to encompass students from diverse age cohorts, thereby facilitating a more comprehensive comparative analysis. Lastly, the variation in sample sizes across family structure and gender categories may introduce bias into our findings. Future studies should consider controlling for these variables to enhance the precision of the results. Future studies could include students from different age groups to enable a more comprehensive comparative analysis. Lastly, although the data were collected from nine provinces, providing a certain breadth in regional distribution, it does not constitute a strict probability sample. Disparities in sample sizes across different family structures and gender categories may introduce bias into the research findings. Future research could validate the findings of this study based on large-scale national surveys.

## Data Availability

The original contributions presented in the study are included in the article/supplementary material, further inquiries can be directed to the corresponding author/s.
